# The effects of social isolation stress and discrimination on mental health

**DOI:** 10.1038/s41398-022-02178-4

**Published:** 2022-09-21

**Authors:** Lasse Brandt, Shuyan Liu, Christine Heim, Andreas Heinz

**Affiliations:** 1 Department of Psychiatry and Psychotherapy, Charité – Universitätsmedizin Berlin (Campus Charité Mitte), Berlin, Germany; 2 Department of Medical Psychology, Charité – Universitätsmedizin Berlin (Campus Charité Mitte), Berlin, Germany

**Keywords:** Neuroscience, Pathogenesis, Psychiatric disorders

## Abstract

Social isolation and discrimination are growing public health concerns associated with poor physical and mental health. They are risk factors for increased morbidity and mortality and reduced quality of life. Despite their detrimental effects on health, there is a lack of knowledge regarding translation across the domains of experimental research, clinical studies, and real-life applications. Here, we review and synthesize evidence from basic research in animals and humans to clinical translation and interventions. Animal models indicate that social separation stress, particularly in early life, activates the hypothalamic-pituitary-adrenal axis and interacts with monoaminergic, glutamatergic, and GABAergic neurotransmitter systems, inducing long-lasting reductions in serotonin turnover and alterations in dopamine receptor sensitivity. These findings are of particular importance for human social isolation stress, as effects of social isolation stress on the same neurotransmitter systems have been implicated in addictive, psychotic, and affective disorders. Children may be particularly vulnerable due to lasting effects of social isolation and discrimination stress on the developing brain. The effects of social isolation and loneliness are pronounced in the context of social exclusion due to discrimination and racism, during widespread infectious disease related containment strategies such as quarantine, and in older persons due to sociodemographic changes. This highlights the importance of new strategies for social inclusion and outreach, including gender, culture, and socially sensitive telemedicine and digital interventions for mental health care.

## Introduction

Mutual interaction and aid are of key importance for socially living animals and humans [1–3]. In humans, prosocial behaviour is especially pronounced and has protective effects on health and adaptation. This behaviour starts early, with infants displaying altruistic attempts to help even foreign persons [2]. It is only at a later stage of individual development that mutual aid is selectively provided only to those persons who may return the favour in the future [3]. Accordingly, social exclusion and isolation are key factors of distress, which have been associated with a wide range of mental disorders including mood disorders, psychosis, and drug dependence [4, 5]. Particularly vulnerable groups for the detrimental effects of social exclusion and discrimination include children, given that traumatization as well as social and physical neglect during early life impact on neurodevelopment and constitute key risk factors for developing mental illness throughout the lifespan [6, 7]. Widespread quarantine regulations during the COVID pandemic have further promoted interest in the behavioural and neurobiological effects of social isolation [8]. In this review, we will (1) summarize neurobiological and behavioural correlates of social isolation in animal models, (2) discuss their effects in humans, including in early life, (3) review current findings regarding effects of particularly salient settings that can cause social exclusion and distress including isolation during quarantine, social discrimination and racism, and loneliness among aging populations, and (4) discuss respective interventions.

## Methods

We performed a narrative review of the literature. The available animal studies were reviewed and synthesized with respect to key effects of social isolation stress. Regarding human findings, the databases PubMed, MEDLINE, and Web of Science were searched from database inception up until May 3, 2022 by one author (L.B.), without restrictions to language or country of origin of the study or publication date (search terms: “social isolation” AND “mental health”). We manually searched references of the included studies and performed additional selective searches with a search engine (i.e., Google scholar). We included original research and reviews focussing on social isolation and mental health. Studies assessing social isolation, loneliness, discrimination, racism, quarantine, older age, or digital interventions concerning social isolation were considered eligible for this review. Studies with human participants and animal models were included and the collected data was synthesized qualitatively.

## Animal models of social isolation distress

### Factors contributing to heterogeneous findings

Regarding animal models, acute versus chronic social isolation effects can be distinguished, which may also differ with respect to age at exposure, and species differences between rodents and primates have to be taken into account [9, 10]. Nevertheless, specific results are often inconsistent, and complex interactions between different types of social stressors have been observed [4]. For example, both long-lasting social isolation [11] and instable social housing conditions that include brief periods of social isolation and changing cage mates [5] increase measures of alcohol seeking and consumption. However, even these two studies vary with respect to rodents (Wistar versus Long Evans rats), age (21 versus 30 postnatal days), procedure (continuing isolation for 60 days versus 1 h isolation per day combined with changing cage mates every 7 days for females and 5 days for males), and assessment of alcohol outcomes (persistence of lever pressing for alcohol in extinction versus alcohol preference when being provided with a free choice between alcohol and water) [5, 11]. A systematic review confirmed substantial variability in protocols applied to assess social instability stress in mice and rats, and suggested efforts to increase standardization and reproducibility of study results [12]. With respect to reproducibility, an intruder paradigm in rodents has been described that reliably induces immune alterations and reflects childhood trauma situations, a key risk factor of mental illness [13], while maternal separation reflects neglect and social isolation stress [14]. For this review, we describe effects of social isolation on key hormonal and neurotransmitter systems in light of key differences in study protocols and discuss their potential relevance for mental disorders.

### Critical periods during neurodevelopment

Critical time periods during neurodevelopment appear to be of specific importance for long-lasting or even irreversible effects and can help to explain some of the inconsistencies in the literature. For example, it has been shown that in mice, early social isolation stress immediately after weaning, i.e., during a period of significant oligodendrocyte maturation, induced altered myelination with loss of oligodendrocyte ErbB3 receptors in the prefrontal cortex and a decreased expression of the ErbB3 ligand neuroregulin-1. Crucially, these alterations did not recover when the mice were replaced into a social environment [15]. In the medial prefrontal cortex, the same post weaning early isolation paradigm altered functional development in a subtype of Layer-5 pyramidal cells [16] and enhanced the activity of inhibitory neuronal circuits [17]. In humans, alterations in white matter tracts were observed in children who grew up in conditions of severe social deprivation [18]. A large body of evidence from human clinical studies provides convincing evidence that an early adverse caregiving environment is associated with multiple profound and long-lived neurostructural, neurofunctional and neurochemical changes at the level of neural circuits that are implicated in the mediation of stress responses and emotion regulation, as well as changes in physiological regulation systems (i.e., the neuroendocrine, autonomic, and immune systems) as well as changes at the molecular level of gene regulation. Together, these changes underlie the manifestation of altered fear learning, heightened stress vulnerability, altered reward processing, and the development of symptoms [19–21]. Discrete sensitive periods for these effects have been suggested, e.g., for effects on the amygdala [20]. Of note, neurostructural changes of the amygdala in severely deprived children are rescued when children are adopted into families before the age of 2 years [19]. Early social isolation stress in non-human primates induced long-lasting reductions in CSF concentrations of the serotonin metabolite 5-HIAA, that were negatively associated with serotonin transporter availability measured in vivo in both adult non-human primates and in humans [22, 23].

### Hypothalamic-pituitary-adrenal (HPA) axis

Altered regulation of the *HPA axis* has long been observed in several serious mental disorders, first and foremost major depression [24]. Studies in persons exposed to childhood trauma revealed sensitization of the HPA axis response to stress, increased central corticotropin-releasing factor (CRF) activity, resistance to glucocorticoids, immune activation, and reduced hippocampal volume, i.e., findings also reported in major depression [25]. Adverse effects of childhood trauma are for example moderated by variations in HPA axis regulatory genes including the FKBP5 and CRH receptor 1 gene [26, 27], and such gene x early environment interactions are mediated by epigenetic changes [28].

Regarding acute stress exposure in persons with mental disorders, a meta-analysis reported a blunted HPA axis response in persons with schizophrenia but not clinical depression [29], while in subjects with alcohol use disorders, a blunted cortisol response was found after physical rather than social stress exposure [30].

Several animal experiments assessed effects of *acute* social isolation on the HPA axis. In mice and rats, a systematic review and meta-analysis suggested that prenatal stress (e.g., due to hypoxia or s.c. dexamethasone injection in the pregnant animal) results in a significant increase in peripheral cortisol concentrations in adult offspring, who also showed increased corticotrophin-releasing hormone levels, particularly in males, and decreased levels of its corticotrophin-releasing hormone receptor 2 [31]. Separating new-born rodents from their mothers and rearing them in social isolation also increases HPA axis activity. Specifically, periodic maternal separation increased basal corticosterone levels [32]. Later, i.e., post-weaning, social isolation rearing with single instead of group housing increased stress-associated glucocorticoid release [32], albeit the latter was only observed in male rodents in one study [33]. *Chronic* social isolation stress increased HPA axis activation in several but by far not all rodent studies, potentially due to differences in age, exact procedures, and duration of social isolation [4, 34].

In non-human primates, findings are more consistent and show that both *acute* and *chronic* social isolation increase basal corticosterone levels [34]. For example, social separation increased corticosteroid levels in plasma in rhesus macaques which were either reared with their mothers or separated from their mothers after birth and reared with peers [35]. These latter monkeys also showed acutely increased levels of serotonin and noradrenalin metabolites in the cerebrospinal fluid (CSF) [36], while dopamine metabolites were reduced [35].

In humans, a meta-analysis showed adverse life events are associated with a small but significant increase in cortisol stored in hair as a marker of HPA axis activity over several months [37]. Regarding stress-associated activation of the HPA axis, a meta-analysis reported that early life adversity was associated with a blunted salivary cortisol response to social stress exposure. In this meta-analysis, effect sizes were higher when adults were compared with children, when maltreatment was compared with other forms of early adversities, and when more female participants were included in the study [38].

Both in animals and humans, early social isolation or deprivation stressors result in neural changes in brain regions relevant to cognitive function, e.g., dendritic loss, reduced synaptic plasticity, and reduced neurogenesis in hippocampus and prefrontal cortex. These effects can contribute to reduced executive functions and facilitate fear learning, generalization, and lack of extinction of fear memories [39].

### Monoaminergic neurotransmission

Alterations in monoaminergic neurotransmission have repeatedly been observed in subjects with major mental disorders. To highlight just a few findings, drug-induced dopamine release has been associated with attribution of incentive salience to drug-associated stimuli [40], while stress-dependent dopamine release may contribute to salience attribution to otherwise irrelevant stimuli in schizophrenia [41]. In humans, acute stress exposure was associated with an indirect measure of dopamine release in subjects with a history of low parental care only [42]. In persons with schizophrenia, meta-analytic evidence confirms that dopamine synthesis capacity, release, and synaptic levels are increased, albeit synthesis capacity was not elevated in treatment-resistant patients, pointing to substantial individual variability [43]. All drugs of abuse release dopamine, preferentially in the nucleus accumbens shell [44]. A (most likely compensatory) down-regulation of dopamine D2 receptors in this brain area was associated with alcohol craving and increased alcohol cue reactivity among detoxified persons with alcohol dependence [45], while during prolonged abstinence, a (rebound) increase in dopaminergic neurotransmission was found [46]. Furthermore, during alcohol detoxification stress in humans, high cortisol levels were associated with a low availability of brainstem serotonin transporters, which in turn correlated with subjective levels of anxiety and depression [23], in accordance with the observation that corticosterone application reduces serotonin transporter density in young adult rats [47].

The non-human primate studies of the group of Dee Higley and co-workers demonstrate how important it is to distinguish between acute and chronic effects of social isolation stress on *monoaminergic neurotransmission*. Specifically, rhesus monkeys who were separated as neonates from their mothers displayed acute increases [35] but chronic reductions in CSF serotonin metabolites [48]. Measuring the available serotonin transporters in both non-human primates and humans revealed inverse correlations between CSF serotonin metabolites and in vivo transporter availability, most likely due to competition between endogenous serotonin levels and radioligand binding at the transporter site [22, 23]. In non-human primates, low serotonin turnover as reflected in low CSF serotonin metabolite levels was associated with a lower level of intoxication by alcohol and increased alcohol intake [22, 49]. In comparison among humans, low serotonin turnover was associated with increased clinical depression among detoxified alcohol-dependent patients [23].

Regarding dopamine, post-weaning social isolation stress in *rodents* did not alter basal extracellular dopamine levels; however, dopamine concentrations in the nucleus accumbens were increased by a depolarizing stimulus, and the effect was stronger in rodents exposed to social isolation [50]. In comparison in humans, using psychostimulants to elicit dopamine release revealed increased striatal dopamine levels among in subjects with schizophrenia at onset of illness, independent of previous neuroleptic medication [51]. Dopamine depletion studies confirmed increased striatal dopamine levels among patients with schizophrenia [52], which were most pronounced in the associative stratum [53]. Stress-induced increases in dopamine release were also found in the associative striatum of patients with schizophrenia [54].

In adult *non-human primates* who were exposed to developmentally early social isolation stress, dopamine receptor sensitivity was increased [55]. In humans, increased dopamine D2 receptor sensitivity was implicated in schizophrenia [56] and may result from neuroleptic medication [57], leading to suggestions of extended dosing [58].

### Glutamatergic and GABAergic neurotransmission

Glutamate and GABA are the main neurotransmitters regulating excitation and inhibition in the human brain [59], as also evinced by human imaging studies [60]. Stress exposure has been suggested to impair neuronal integrity of excitatory glutamate neurons and inhibitory GABA interneurons in limbic and cortical brain areas also implicated in major affective disorders [61]. Due to the acute therapeutic effects of ketamine application, glutamate function has been in the focus of discussion regarding pharmacological treatment of major depression [62]. Indeed, meta-analytic evidence from human spectroscopy studies suggest decreased glutamate and glutamine levels in the medial prefrontal cortex of persons in major depression, although results were only found in medicated patients [63]. Glutamate and specifically NMDA receptor dysfunction have also been implicated in schizophrenia [64] and in addictive disorders, for example, alcohol dependence [65].

In *rodents* exposed to post-weaning social isolation stress, a depolarizing stimulus increased glutamate levels, while this was not found in individuals not exposed to social isolation [50]. Generally, social isolation stress tends to increase glutamatergic neurotransmission and inhibit GABAergic effects [4]. In young *non-human primates* living with their mothers, application of an inverse benzodiazepine agonist induces anxiety and increases CSF dopamine and noradrenaline metabolites, and the effect on noradrenaline metabolites was further increased by acute maternal separation [66].

Altogether, effects of social separation stress have repeatedly been shown to activate the HPA axis and interact with monoaminergic, glutamatergic, and GABAergic neurotransmitter systems, inducing long-lasting reductions in serotonin turnover and alterations in dopamine receptor sensitivity in non-human primates. These findings are of particular importance for human social isolation stress, as the same neurotransmitter systems have repeatedly been implicated in addictive, psychotic and affective disorders [10, 67, 68]. Hence, it is conceivable that social isolation, discrimination and COVID-related quarantine regulations may have profound and lasting impact on mental health, necessitating strategies for intervention and prevention in vulnerable groups.

## Social isolation and discrimination in humans

### Social isolation and loneliness

Social isolation is a growing public health issue associated with poor physical as well as mental health outcomes including increased morbidity and mortality and reduced quality of life [69–72]. Social isolation can be defined as an objective lack of social interactions while the related concept of *perceived* social isolation (e.g., loneliness and perceived social support) is characterized by a subjectively perceived lack of social interactions [70, 73]. Loneliness is specifically defined as the distressing experience of a discrepancy between one’s desired and actual social connection [74, 75]. It is important to note that the quality of social interactions mediates between the objective and subjective dimensions of social isolation, which can alter the direction and strength of association between social isolation and loneliness. For example, an individual is at risk of loneliness when the quality of the social interactions is invalidating or discriminating [73]. Accordingly, social exclusion, discrimination, and racism can cause social isolation stress and were implicated in the manifestation of several mental disorders including schizophrenia [76–79]. In comparison, solitude has been conceptualized as voluntary distancing from social networks, whereas loneliness is considered to be involuntary and characterized by a desire of relationships [80].

Social isolation and loneliness appear to be widespread and growing phenomena with about a third of the population in industrialized countries being affected by perceived social isolation [71], albeit prevalence rates vary due to psychosocial and cultural differences between populations [73]. For more than 40 years, the effects of social isolation on human physical and mental health have been a focus in social neuroscience [73]. While research in the 1980s focused more on effects of social control through social networks, more recent research aimed to unravel the psychological, behavioural, and biological pathways such as the neuroendocrine pathways of social isolation including activation of the HPA axis and sympathetic adrenomedullary (SAM) axis [73]. The neuroendocrine, neural, and behavioural responses to social isolation can lead to negative physical and mental outcomes [73, 81]. It has been hypothesized that such effects may not primarily interfere with short-term survival of the isolated individuals, but instead lead to increasingly negative effects over time [73, 81]. For example, social isolation can increase alertness to potential harm from other individuals accompanied by anxiety and depressive symptoms, age-related cognitive deficit, hostility, and social withdrawal, and may alter impulse control, pronounce sleep fragmentation, and increase vascular resistance [73, 81]. The association between loneliness, social isolation, and inflammation (e.g., C-reactive protein, fibrinogen, and Interleukin-6) has been investigated in a recent meta-analysis, however, there is substantial heterogeneity in the original trials and the effects are currently inconsistent [82].

The *epidemiological* association between social isolation and negative mental health outcomes such as depression is established but largely based on observational studies with limited evidence of causality (e.g., social isolation may precede depression or vice versa and one can augment the other) and from populations in the northern hemisphere [70]. In 2017, more than 40 systematic reviews and meta-analyses had been published assessing the effects of social isolation and loneliness on public health [70]. A meta-analysis of cohort studies indicated an increased probability of mortality associated with both social isolation (odds ratio 1.29; 95% CI 1.06–1.56) and loneliness (odds ratio 1.26; 95% CI 1.04–1.53) [83]. This finding did not suggest a large difference in effect size between social isolation on the one hand and loneliness on the other, but potential differences remain to be determined in prospective studies [83]. Among physical health outcomes, the strongest evidence of an association with social isolation and loneliness has been reported for cardiovascular disease including hypertension, cardiovascular risk, and postmyocardial infarction mortality [70, 84, 85]. For example, a meta-analyses of prospective studies showed an increased relative risk of cardiovascular disease including coronary heart disease (relative risk 1.5; 95% CI 1.2–1.9) among adults experiencing social isolation [84].

The evidence of associations between social isolation and loneliness and the mental health outcomes well-being (for social isolation and loneliness), depression (for social isolation), suicide (for social isolation and loneliness), and dementia (for loneliness), has been determined to be moderately strong using the so-called “grading of recommendations, assessment, development and evaluations” (GRADE) approach, which is a systematic approach of grading the certainty of evidence [70]. An older meta-analysis from 2000, which included 286 empirical studies, indicated that the quality of social contacts showed a stronger association with well-being than the quantity of social contacts [86]. This finding suggests a mediating effect of the quality of social interactions for mental health outcomes such as well-being. In a systematic review from 2015, larger and more diverse social networks (i.e., diversity defined according to the social network composition consisting of family members, friends, and co-workers) were associated with a protective effect against depression in the general population, including individuals with chronic physical illness [87]. In agreement, another systematic review concluded that the quality of social relations had a greater impact on the development of depression in late life than the quantity [88]. The risk of post-stroke depression has also been associated with post-stroke social isolation [89]. In individuals with multiple sclerosis, social isolation was determined as a risk factor of suicidal ideation based on a systematic review including 12 studies [90]. Also, the sense of belonging can be reduced due to social isolation and loneliness, and was weakly associated with suicidality in a systematic review from 2013 [91].

Loneliness was moderately associated (r .32, 95% CI 0.20–0.44) with psychosis as indicated in a recent meta-analysis [92]. Epidemiological assessments on neighbourhood- or area-level indicated an increased risk of psychotic disorders for social isolation and social fragmentation [93, 94]. However, a systematic review from 2018 highlighted contradictory findings regarding the association between loneliness and psychotic symptoms, which can be influenced by psychosocial factors such as depressive symptoms, anxiety, social cognition deficits, poor social support, stigma, and perceived discrimination [95].

Clinical observations of an association between alcohol use and loneliness have been described even before the 1950s [96]. These observations are supported by a systematic review from 2020, which shows that individuals with substance use are at increased risk of loneliness compared to the general population, potentially due to social stigma and other difficulties maintaining relationships [69]. The association between substance use and loneliness appeared to be pronounced in younger people and women, but the reasons remain uncertain due to the correlational results [69]. The prevalence of alcohol and tobacco use was also associated with psychosocial factors including loneliness in a systematic review focusing on Brazilian adolescents from 2012 [97]. Social isolation can contribute to an increased alcohol consumption and maintenance of alcohol use disorder. Individuals who lost their work may be at particular risk of increased alcohol consumption in case of loneliness and pre-existing high levels of alcohol consumption [10].

The impact of social isolation stress may particularly be pronounced due to isolation and quarantine during the COVID-19 pandemic, in relation to discrimination and racism, and in association with older age, which are all instances with an increased risk of loneliness and social isolation [98–100]. The following sections will focus on social isolation in the context of these instances.

### Discrimination and racism

Discrimination is defined as making distinctions between people based on prejudice and biologically unjustified assumptions of categorical differences attributed to perceived “race” or “ethnicity” [99, 101–103]. Racism discriminates according to scientifically disproven and discredited concepts of “racial classifications” and is not limited to individual beliefs, values, and interactions, but also manifests on a systemic level such as structural racism, which refers to racial discrimination in housing, education, employment, income, health care, justice, and media, among other societal domains [99, 101, 102]. Racism on the level of personal beliefs and structural racism act mutually reinforcing with devastating individual and societal consequences, which are also evident in the field of mental health [99, 104–107]. A meta-analysis from 2015 indicated an association of racism and poorer mental health (r −0.23; 95% CI −0.24, −0.21) such as depression, anxiety, and psychological stress [77]. The effect size was twice as large for the association between racism and poorer mental health compared to racism and poorer physical health (r −0.09; 95% CI −0.12, −0.06; especially overweight-related outcomes such as increased BMI) [77]. An older meta-analysis from 2009 had reported similar effects for mental and physical health, which may have been related to a broader assessment of perceived discrimination compared to the narrower focus on “race” and classifiers such as ethnicity and nationality in the review from 2015 [77].

It has been hypothesized that the increase in adverse mental health and physical outcomes may neuroendocrinologically be associated with HPA axis dysregulation as a response to stress as well as vigilance induced by discrimination and racism [77, 106, 108]. The adverse mental health effects have an early onset in children and adolescents, as there have been consistent reports of positive associations between racist discrimination and anxiety as well as depression, and of negative associations between racist discrimination and self-esteem as well as self-worth [109]. The impact of discrimination and other potentially traumatic experiences associated with migration [76, 110–112] and childhood adversities [113] on the development of serious mental illness including psychotic disorders has repeatedly been addressed, and a recent umbrella review [114] demonstrated a strong effect of racist discrimination (odds ratio 3.90; 95% CI 3.25, 4.70), childhood adversities (odds ratio 2.81; 95% CI 2.03, 3.83) and migration (odds ratio 2.22; 95% CI 1.75, 2.80) on the risk of developing a non-affective psychotic disorder. Discrimination and a lack social support can also contribute to the increased risk of psychosis for individuals with a visible minority status (e.g., African and Caribbean communities in England) [113–115]. This effect may be particularly strong when there is perceived social isolation due to low community support from equally afflicted persons, e.g., due to so-called low “ethnic density” [116]. Such effects of local social support have also been described with respect to affective disorders, however, the effect of ethnic density on depression appeared more heterogenous, possibly due to potentially confounding effects of local poverty and socioeconomic disadvantage [117–119].

Exposure to discrimination and racism can be associated with personal insecurity in social interactions [76], and ambivalent and ambiguous social interactions can in turn be accompanied by increased anxiety, vigilance, and feelings of threat, resulting in an increased focus on the current situation [108, 114]. On a neurobiological level, imprecise prior knowledge of the expected outcome and a stronger reliance on current sensory input increases prediction errors, which can trigger dopamine release and increase the risk of developing a psychotic disorder [76, 120–122].

Migrant status, economic disadvantage, and social exclusion can lead to social isolation, which highlights the vulnerability of certain groups such as refugees with impaired possibilities of social participation and income [110, 119]. A meta-analysis of first- and second-generation migrants demonstrated an interaction between minority status, social inequality, and increased mental disorders, and highlighted the negative impact of downward social mobility and underemployment across generations of vulnerable persons with migrant status [123]. Regional effects such as neighbourhoods with pronounced poverty and heightened risk of social exclusion are associated with increased risk of mental health disorders, and the association does not appear to be explained by general urbanization [113, 117–119].

These findings have implications for mental health intervention strategies and policies. Strategies and policies aiming to mitigate the mental health care burden (especially for vulnerable groups with migrant status, socioeconomic disadvantage, and risk of social exclusion and isolation) require to focus on reducing poverty and income inequality and promoting opportunities of social participation, work with fair income, and access to mental health care with intercultural competences, in addition to fighting discrimination and providing mental health care for the general population [119, 124, 125]. Practical guidance on how to engage in strategies to reduce discrimination and racism in clinical practice have been advocated by health care organizations such as the Royal College of Psychiatrists [126], the German Association for Psychiatry, Psychotherapy and Psychosomatics [127], the European Psychiatric Association [103], and the American Academy of Pediatrics [128].

### Quarantine and isolation

The ongoing COVID-19 pandemic has led to a drastic increase in infectious disease containment strategies such as quarantine and isolation as well as other measures of physical distancing [129–131]. These strategies are intended to reduce the spread of an infectious disease: quarantine typically refers to restricted movement and limited close interactions for individuals who have been exposed to contagious persons, while isolation refers to similar measures for individuals who are infected [131–133]. The containment-related restrictions have adverse effects on mental health such as increased anxiety, depression, and stress-related disorders, and can exacerbate substance use disorders, psychotic disorders, anger, and domestic violence [8, 98, 129, 134]. A recent meta-analysis showed increased prevalence rates of psychological morbidities including poor sleep quality (40%), stress (34%), psychological distress (34%), insomnia (30%), post-traumatic stress symptoms (27%), anxiety (26%), and depression (26%) amidst the COVID-19 pandemic [135]. Children and adolescents may also experience increased rates of depression and anxiety during and after containment-related isolation based on a recent rapid systematic review (including studies with high risk of bias) [136].

A retrospective cohort study of over 62,354 COVID-19 cases in the US suggested a bidirectional association between COVID-19 and psychiatric disorders: individuals with a history of COVID-19 appeared to be at increased risk of mental health disorders, particularly anxiety disorders, insomnia, and dementia, and conversely, a history of a psychiatric diagnosis might be a risk factor for an infection with COVID-19, but confounding by socioeconomic factors could not be excluded [134]. Minorities have been strongly affected by COVID-19 in the United States, and minority status has been associated with increased insecurity regarding money for food and rent among women during the pandemic [137], however, no significant differences were observed among older adults regarding mental health or stress [138].

Adverse effects of containment strategies are established especially for psychosocially vulnerable individuals with mental disorders [8, 129]. The increased risk of adverse mental health effects for individuals with such disorders persisted even after correction for levels of psychological outcomes at baseline [139, 140]. Socioeconomical determinants of negative mental health effects of containment may be financial loss and lower levels of income and education, which were associated with depression, anxiety, anger, and stress-related disorders [141–146]. Fewer social resources before containment (e.g., lower levels of social capital, perceived social support, and neighbourhood relationships) were also associated with more negative mental health effects such as depression and anxiety during containment [145, 147, 148]. The association between low income and loneliness with depression and anxiety is especially critical during pandemic-related social isolation, income disparity, and loss of employment [146, 149]. Health care workers can be affected by social stigma in addition to the risk of infections due to their potential exposure to contagious patients and may be another group with higher probability of negative psychological outcomes [8]. In a recent meta-analysis, the evidence of effects of containment were heterogenous for the first one to three days of containment, but the adverse effects became more consistent for containment over one to two weeks [8]. For example, anger was a psychological outcome that could increase over the course of containment, which has social implications such as potentially increasing domestic violence, thus highlighting the importance of maintaining access to child care facilities and other institutions that support children, adults, and families in distress [141, 150–152]. Stress-related symptoms can persist over years, but more longitudinal studies are needed to elucidate effects persisting beyond weeks to months [8]. It has been debated whether adult individuals who are socioeconomically less vulnerable (e.g., not lacking financial and social resources) might in the short-term experience even reduced social stress through quarantine measures, but more research is required to assess predictors of positive outcomes [129].

Several measures have been recommended aiming to mitigate the adverse mental health effects associated with social isolation during the pandemic and especially quarantine: it appears advisable to keep the duration of the containment as short as possible (and as long as needed from an infectious disease standpoint), providing people with as much information as possible (e.g., rapid and reliable information regarding rationale for the containment and the planned duration), and improving communication such as availability of digital communications to maintain the social network [98, 136]. Vulnerable groups such as individuals with mental disorders require special attention including continued access to psychiatric care and other mental health services [98]. Large-scale research strategies are currently being implemented with the aim of comprehensively assessing the rapidly growing research data related to the COVID-19 pandemic such as meta-ecological approaches and crowdsourcing [129, 130], which may further improve the evidence of mental health effects associated with the pandemic and social isolation.

### Social isolation and older age

Social isolation and loneliness in older persons is a rapidly increasing problem [100]. Global demographic changes are transforming the age structures of societies and leading to expanding segments in the higher age groups [153, 154]. Decreasing birth rates and higher life expectancies are among the drivers of this ongoing trend [154]. Globally, 727 million people were in the age group of above 64 years in 2020, and the number of people in this age group are expected to more than double by 2050 [153]. The relative increase in this age group is substantial and will grow from 9.3% in 2020 to 16% in 2050 [153]. Countries in Western Europe, the United States, and Japan appear to be especially affected, while many countries in the Global South such as Nigeria and Uganda have pronounced younger age structures with large proportions of the population under the age of 30 years [154].

Social isolation and loneliness are not only relevant due to an increasing number of people with older age, but also because of an increasing risk of social isolation and loneliness associated with changes in family structures and living arrangements such as size and composition of households, resulting in growing proportions of older individuals who are living in single or smaller households [153]. The prevalence rates of loneliness are high in older age groups, with estimates between 25 and 29% among community-dwelling individuals in the US aged above 69 years; similar rates are reported in European countries and China, albeit the methodological heterogeneity appears to be substantial [80]. Living in residential care has also been associated with increased social isolation and loneliness [155–157].

The neurobiological and endocrinological effects of social isolation and loneliness combined with isolation-related adverse health behaviours (e.g., increased alcohol use and smoking and decreased physical activity and poorer nutrition), as well as sleep disturbances (e.g., shorter duration of sleep and daytime fatigue), could exacerbate the risk of physical and mental health disorders, with particularly detrimental effects in physically and mentally vulnerable populations such as older persons [80]. The mutually reinforcing causal links between social isolation, loneliness, physical health, and mental health appear to have a strong impact in the older age groups [100]. A systematic review from 2014 indicated that suicidal thoughts are common among older individuals in long-term care facilities, with prevalence rates ranging between 5 and 33% and correlating positively with depression, social isolation, loneliness, and functional decline [158]. This finding is in agreement with another systematic review from 2012, which showed an inverse association between social connectedness (e.g., with family, friends, and social groups) and suicidal behaviour (i.e., suicidal ideation, non-fatal suicidal behaviour, and suicide in later life) based on an analysis of original trials with participants aged 65 years and older [159]. Social isolation is not only associated with affective symptoms and suicidal behaviour but also appears to be linked to an increased risk of cognitive decline and dementia [70]. A systematic review from 2015 indicated that loneliness could be negatively correlated with global cognitive function, test results regarding the so-called intelligence quotient, processing speed, immediate recall, and delayed recall, however, the effects were partly reduced after controlling for demographic and psychosocial factors [160]. A meta-analysis of 19 longitudinal studies showed an pronounced risk of incidence of dementia for increased loneliness (relative risk 1.58; 95% CI 1.19–2.09), lower social participation (relative risk 1.41; 95% CI: 1.13–1.75), and less frequent social contact (relative risk 1.57; 95% CI 1.32–1.85) [161]. A review from 2016 suggested that loneliness could be associated with impaired daytime functioning, reduced physical activity, lower subjective well-being, and poorer physical health, and that loneliness could prospectively predict increased depressive symptoms, impaired cognitive performance, dementia progression, likelihood of nursing home admission, and somatic outcomes such as hypertension, heart disease, and stroke [80].

Regarding biological pathways linking loneliness, cognitive function, and mental health in older age groups [100, 160], it has been suggested that loneliness could be associated with prolonged activation of the hypothalamus-pituitary-adrenal (HPA) axis and increased inflammation [160]. HPA-axis dysregulation and increased cortisol may contribute to poorer overall cognitive functioning, episodic memory, executive functioning, language, spatial memory, processing speed, and social cognition [162, 163]. The hippocampus is an important limbic region for executive functions and memory formation and increased cortisol levels might lead to oxidative stress and dysregulation and atrophy of the hippocampus [163]. In addition, loneliness can cause psychological distress, which in turn can induce vegetative and inflammatory responses [164]. Increased inflammation has been suggested to play a role in the development of dementia, especially Alzheimer’s dementia [165, 166]. The relationship between loneliness and mental illness includes negative reciprocal effects, i.e., loneliness can have a negative impact on mental illness and mental illness may have a negative impact on loneliness [100].

Altogether, depression and impaired cardiovascular health are among the most often researched outcomes in relation to social isolation and loneliness among persons with older age [100]. An important future direction of research is to further investigate the role of the cultural and socioeconomical context to improve our understanding of loneliness as a risk factor of mental illness in older individuals [160]. It is relevant to note that the growing public health issue of mental health problems among older adults can be further accentuated by quarantine measures and physical distancing during the ongoing COVID-19 pandemic [167]. This highlights the need of interventions to specifically address social isolation and loneliness in vulnerable persons with older age in addition to interventions targeting the general population [167].

## Social isolation and discrimination interventions

Interventions designed to reduce social isolation stress involve two distinct concepts: subjective social isolation (including loneliness and perceived social support) and/or objective social isolation (having little social contact with other people measured by social network size or the frequency of social contacts with others) [168]. Furthermore, social exclusion, discrimination, and isolation are to be addressed at an institutional level [103, 126–128].

On an individual or micro-level, promising interventions targeting subjective and objective social isolations may include changing maladaptive or negative cognitions for subjective social isolation, and using mixed-methods strategies and designing supported socialization programmes for objective social isolation [168]. A meta-analysis of interventions to ease social isolation stress [169] has identified and categorized four core intervention strategies including: (1) improving social skills (e.g., friendship enrichment programme, family psychoeducation), (2) enhancing social support (e.g., peer support and social creation groups, human-human interaction or human-robot interaction scenarios within the context of the confidant relationship, animal-assisted therapy, increasing social forms of video gaming), (3) increasing opportunities for social contact (face-to-face or online meetings, social prescribing service, asset-based community development), and (4) changing maladaptive social cognition (cognitive-behavioural therapy, self-help or reminiscence therapy groups). Among those four intervention strategies, the most effective one in reducing social isolation stress appears to address maladaptive or negative social cognition by changing social behaviour and thereby building social connections [169–171].

To reduce social exclusion and discrimination at individual, community, and population levels, previous reviews evaluated the effectiveness of interventions that focused on knowledge, attitudes, and behavioural outcomes [172, 173]. These three outcomes refer to three respective domains: ignorance in the cognitive domain, prejudice in the affective domain, and discrimination in the behavioural domain [174–176]. To reduce discrimination, social contact interventions have been found to be effective in increasing knowledge (reduce ignorance in the cognitive domain) and improving attitudes (reducing prejudice in the affective domain) in the short term, but there is lesser evidence for long-term benefits [172, 173]. The wide variation in results may arise from differences in the intervention intensity and focus (often mainly on increasing knowledge and less on changing attitude) or using different types of methodologies [172, 173]. The interventions that aimed at increasing knowledge, attitudes, and intended behaviour yielded only small-to-moderate effect sizes [172, 173, 177]. Accordingly, public information campaigns against racism or stigma related to mental disorders need to provide personal contact [178, 179], while relying on the provision of genetic and biological information to explain mental illness may have unintended and unwanted effects including increasing social distance and stigmatization [180–182].

The COVID-19 pandemic has forced a worldwide lockdown, with people confined to their homes and burdened by quarantine and isolation [8, 146, 183]. In this context, interventions had to be designed for remote implementation instead of face-to-face interactions at an individual as well as community level. The COVID-19 pandemic thus highlighted the importance of using digital interventions that reach a broad audience in order to prevent or reduce social isolation stress and discrimination [183–187]. Remote interventions can be achieved through self-guided programmes or delivered by community health workers or mental health professionals via online social media channels (e.g., direct message exchange) or traditional communication channels (e.g., telephone or video conversation, especially for older adults with limited digital literacy) [167]. For example, an online relaxation intervention including guided breathing and mindfulness body scan exercises and listening to rain and water sounds, which can lessen COVID-19-related stress during social isolation [188]. Home-based physical exercise and cognitive training programmes can lead to synergistic effects regarding the respective benefits associated with physical and mental health [189]. However, individuals’ preference for communication methods, their pre-pandemic digital literacy, and their acceptance and attitude towards online support need to be considered when designing and implementing interventions [167, 184]. Remote digital-based interventions may offer mental health and psychosocial support in a more timely and cost-effective manner to those who feel isolated or discriminated against [184]. Group peer support can promote recovery, and integrating peer support into digital interventions can effectively reduce clinician burden, not only in times when in-person social interactions are restricted [184].

On the macro-social level, community-based intervention can mitigate social isolation stress, and discrimination by using peer support and community empowerment for building skills and sharing knowledge [190]. A participatory approach has been identified as key component for empowering individuals and communities to actively take part in the intervention design and development processes that address their social and mental health needs [190]. Accordingly, bringing together university students and recently resettled refugees for mutual learning and mobilization of community resources can help to reduce mental health disparities [191]. Further reductions in mental health disparities can be achieved by increasing the perceived personal relevance of interventions that improve engagement in mental health care for people from diverse backgrounds [192, 193].

Regarding the methodological quality of such intervention studies, previous reviews emphasized the need for using robust randomization, for detecting invalid test and scale scores, and for measuring the effectiveness of interventions at long-term follow-up beyond the immediate post-intervention period [194–196]. Evaluations of the quality of interventions indicated that they often lack fidelity (i.e., adherence to the planned study design, training, delivery, receipt, and enactment) [177, 197, 198] and a theoretical or conceptual framework [194, 195]. There is also a lack of research in low or middle-income countries [172], insufficient evidence on the costs and/or cost-effectiveness of interventions [194], and a need for more studies on multi-exposure, multi-component, and long-term interventions [194, 196].

Altogether, our review refers to the biological mechanisms implicated in the effects of social isolation and discrimination on mental disorders, highlights their clinical relevance, and suggests interventions on the individual and community level. Current major social and environmental challenges such as climate change and population displacement, pandemics, and an aging population in industrialized high-income countries all increase the risk of social isolation and exclusion and emphasize the need for targeted interventions including digital technologies to reach a broad public.
